# Anti-cytomegalovirus IgG antibody titer is positively associated with advanced T cell differentiation and coronary artery disease in end-stage renal disease

**DOI:** 10.1186/s12979-018-0120-0

**Published:** 2018-07-02

**Authors:** Feng-Jung Yang, Kai-Hsiang Shu, Hung-Yuan Chen, I-Yu Chen, Fang-Yun Lay, Yi-Fang Chuang, Chien-Sheng Wu, Wan-Chuan Tsai, Yu-Sen Peng, Shih-Ping Hsu, Chih-Kang Chiang, George Wang, Yen-Ling Chiu

**Affiliations:** 1 Graduate Institute of Clinical Medicine, College of Medicine, National Taiwan University, Taipei, Taiwan; 2 Department of Internal Medicine, National Taiwan University Hospital Yun Lin Branch, Douliu, Taiwan; 3 Department of Internal Medicine, Far Eastern Memorial Hospital, New Taipei City, Taiwan; 4 Graduate Institute of Immunology, College of Medicine, National Taiwan University, Taipei, Taiwan; 5 Institute of Public Health, School of Medicine, National Yang Ming University, Taipei, Taiwan; 6 Department of Medicine, National Taiwan University Hospital, Taipei, Taiwan; 7 Biology of Healthy Aging Program, Division of Geriatric Medicine and Gerontology, Johns Hopkins University School of Medicine, Baltimore, MD USA; 8 Graduate Program in Biomedical Informatics, Yuan Ze University, Taoyuan City, Taiwan

**Keywords:** Cytomegalovirus, End-stage renal disease, Cardiovascular disease, Immunosenescence, Immunology

## Abstract

**Background:**

Accumulating evidence indicates that persistent human cytomegalovirus (HCMV) infection is associated with several health-related adverse outcomes including atherosclerosis and premature mortality in individuals with normal renal function. Patients with end-stage renal disease (ESRD) exhibit impaired immune function and thus may face higher risk of HCMV-related adverse outcomes. Whether the level of anti-HCMV immune response may be associated with the prognosis of hemodialysis patients is unknown.

**Results:**

Among 412 of the immunity in ESRD study (iESRD study) participants, 408 were HCMV seropositive and were analyzed. Compared to 57 healthy individuals, ESRD patients had higher levels of anti-HCMV IgG. In a multivariate-adjusted logistic regression model, the log level of anti-HCMV IgG was independently associated with prevalent coronary artery disease (OR = 1.93, 95% CI = 1.2~ 3.2, *p* = 0.01) after adjusting for age, sex, hemoglobin, diabetes, calcium phosphate product and high sensitivity C-reactive protein. Levels of anti-HCMV IgG also positively correlated with both the percentage and absolute number of terminally differentiated CD8+ and CD4+ CD45RA+ CCR7- T_EMRA_ cells, indicating that immunosenescence may participate in the development of coronary artery disease.

**Conclusion:**

This is the first study showing that the magnitude of anti-HCMV humoral immune response positively correlates with T cell immunosenescence and coronary artery disease in ESRD patients. The impact of persistent HCMV infection should be further investigated in this special patient population.

**Electronic supplementary material:**

The online version of this article (10.1186/s12979-018-0120-0) contains supplementary material, which is available to authorized users.

## Background

Human cytomegalovirus (HCMV), a member of the β-herpesvirus family, contains a double-stranded DNA genome and persists in certain host cells indefinitely after primary infection [1]. Worldwide HCMV seroprevalence among women of reproductive age is about 45–100% [2]. Overall seroprevalence rate increases with age [3], reaching above 60% among people older than 50 and is considerably higher in Asian countries [4, 5]. Importantly, in addition to causing opportunistic infections, congenital infections, and mononucleosis, HCMV infection also possess long-term threats to immunocompetent individuals. Mounting evidence strongly suggests the implication of persistent HCMV infection in autoimmunity, cancer, poor response toward vaccination, cardiovascular disease and mortality [6–8].

Large-scale epidemiological studies and meta-analysis have suggested an association between HCMV seropositivity and cardiovascular disease [9, 10]. Earlier observations noted that HCMV seropositivity is associated with restenosis after coronary atherectomy [11]. HCMV can infect endothelial cells, which explains why its viral DNA is often found at sites of arterial disease [12]. HCMV could also contribute to cardiovascular disease via indirect mechanisms such as inducing systemic inflammation, cytokine release [13], and an increase in blood pressure [14]. Several studies based on HCMV-specific IgG titers also showed that higher IgG titers against HCMV, but not antibodies against herpes simplex virus 1, are significantly associated with incident coronary artery events [15, 16]. Since HCMV IgG increases in individuals with virus shedding [17] and its level positively correlates with HCMV-specific IgM [18], an increase of HCMV IgG during latency might indicate frequent or recent virus reactivation.

Of note, HCMV infection also leads to an imbalance of the T cell homeostasis, causing significant loss of naïve CD8+ T cells, accumulation of terminally differentiated memory CD4+ and CD8+ cells – premature aging of T cells, also known as “immunosenescence” [19, 20]. This perturbation of immune system not only causes impaired vaccine response [21], but also poor survival [22]. These terminally differentiated memory T cells might be preferentially recruited to vascular endothelium via upregulation of CX3CR1 and participate in the process of atherosclerosis [23, 24].

Cardiovascular mortality is the most important cause of death in ESRD (end stage renal disease) patients worldwide [25, 26]. ESRD patients exhibit a striking 20–400 fold higher risk of cardiovascular death compared to age-matched healthy individuals. However, traditional cardiovascular disease risk factors only explain a relatively small proportion of such high cardiovascular disease burden [27]. Similar to healthy individuals, CMV seropositivity is related to the amount of terminally differentiated T cells in patients with ESRD [28, 29]. To our knowledge, no study has attempted to study the relationship between the titer of HCMV-specific IgG with either immunosenescence or atherosclerotic heart disease in ESRD. We thus established the “immunity in ESRD” (iESRD) study, which is an ongoing longitudinal study aiming to investigate the impacts of immunological mechanisms on cardiovascular outcome in ESRD patients. The current study reports the association between HCMV IgG titer with immunosenescence and cardiovascular co-morbidities based on the baseline data from this cohort study.

## Methods

### Participants

The immunity in ESRD (iESRD) study is a cohort study investigating the effect of immunological factors on outcomes of hemodialysis patients. Patients and healthy controls were recruited from Far Eastern Memorial Hospital and National Taiwan University Hospital Yun Lin Branch. Far Eastern Memorial Hospital is located in New Taipei City in northern Taiwan and National Taiwan University Yun Lin Branch is located in southern Taiwan. A total of 432 patients signed informed consent to join the study and were screened for eligibility. Those with recent hospitalization within three months, active or chronic infection requiring antibiotics, incomplete blood test results or poor blood samples quality were excluded, resulting in 412 patients enrolled in the iESRD study (198 from Far Eastern Memorial Hospital and 214 from National Taiwan University Hospital). Among these patients, 408 were CMV seropositive and were analyzed in the current study. Most patients (99.5%) received hemodialysis at least 4 h per session, thrice a week; only two patients underwent twice a week hemodialysis. All patients were treated by their primary care nephrologists according to the Kidney Disease Outcomes Quality Initiative (KDOQI) guidelines.

### Data collections and laboratory exams

Blood samples were collected before the start of hemodialysis sessions in the middle of week. In addition to hematological and biochemical tests, peripheral blood mononuclear cells (PBMC) were sampled. Intact-parathyroid hormone (i-PTH) immunoradiometric assay (Cisbio) and high sensitive C-reactive protein (hs-CRP) nephelometry (Siemens) were also tested. By history taking and detailed chart reviews, baseline co-morbidities and clinical laboratory data were recorded. HCMV-specific IgG titer was measured by ELISA (Roche Elecsys assay) at Far Eastern Memorial Hospital. Plasma level of inflammatory cytokines were measured using the human IL-6 Quantikine HS ELISA kit and human TNFα Quantikine ELISA Kit from R&D systems.

### Immunophenotyping and multicolor flow cytometry

After blood collection, peripheral blood mononuclear cells (PBMCs) were immediately isolated by Ficoll-Paque PLUS gradient centrifugation following the manufacturer’s protocol (GE Healthcare). For flow cytometry analysis, briefly, singlets were collected by forward scatter area and height. CD3-AF700 (clone UCHT1, BioLegend) was used to identify CD3+ T cells from the lymphocytes gated by forward and side scatter properties. CD4+ and CD8+ T cells were determined by CD4-PerCP-Cy5.5 and CD8-APC-Cy7 (clone OKT4 and SK1, BioLegend) respectively, and the T cell differentiation states were determined by CCR7-APC, CD45RA-Alexa488 (clone G043H7 and HI100, BioLegend) and CD28-PE-Cy7 (clone 28.2, eBioscience).

Monocyte staining was performed according to previous studies performed by Zawada et al., which indicated that a pan-monocyte marker such as CD86 is necessary to correctly enumerate monocyte subsets [30]. After gating on the forward scatter/side scatter, monocytes were determined by expression of CD86-PE (clone IT2.2, eBioscience), and were further classified as classical (CD14++CD16-), intermediate (CD14++CD16+), and non-classical (CD14 + CD16++) by CD14-FITC and CD16-APC (clone M5E2, Biolegend and clone 3G8, eBioscience). In general, the percentage calculated for a specific immune cell subset refers to the percentage of a specific cell subset among the mother population on flow cytometry.

### Cardiovascular co-morbidities

Medical comorbidity status of all patients were determined by careful review of medical history and radiological reports. The documented coronary artery disease (CAD) was defined as either 1) > 50% stenosis of at least one coronary artery on coronary angiography or 2) documented perfusion defect(s) on stressed cardiac nuclear scan. Cardiovascular disease (CVD) was defined as having documented CHF, CAD, stroke and/or peripheral arterial disease (PAOD). Congestive heart failure (CHF) was diagnosed clinically as a syndrome in which patients have symptoms and signs resulting from an abnormal cardiac structure or function by cardiologists.

### Statistical analyses

Patient characteristics were described as mean ± standard deviation for continuous variables and frequency for categorical variables. These variables were analyzed by ANOVA and Chi-square test, respectively. Pearson correlation was applied to evaluate the correlation of log-transformed HCMV-specific IgG with immune cell subsets frequencies and absolute cell numbers.

Binary and ordered logistic regression models were used to calculate the predictive value of HCMV IgG level on cardiovascular co-morbidities. Univariate logistic regression was performed to calculate the *p* for trend value for determining the relationship between HCMV IgG quintiles and individual medical co-morbidity. Multivariable-adjusted logistic regression models, including age, gender, diabetes mellitus, albumin, hemoglobin, calcium phosphate product and high sensitivity-CRP were used to investigate the association between HCMV IgG level and co-morbidities. All statistical tests were two-tailed, and a *p* value of less than 0.05 was considered be significant. The statistical analyses were performed with SPSS Version 25 (IBM) and STATA version 14.2 (StataCorp).

## Results

### HCMV IgG levels in ESRD patients are elevated compared to control individuals

**Fig. 1:**
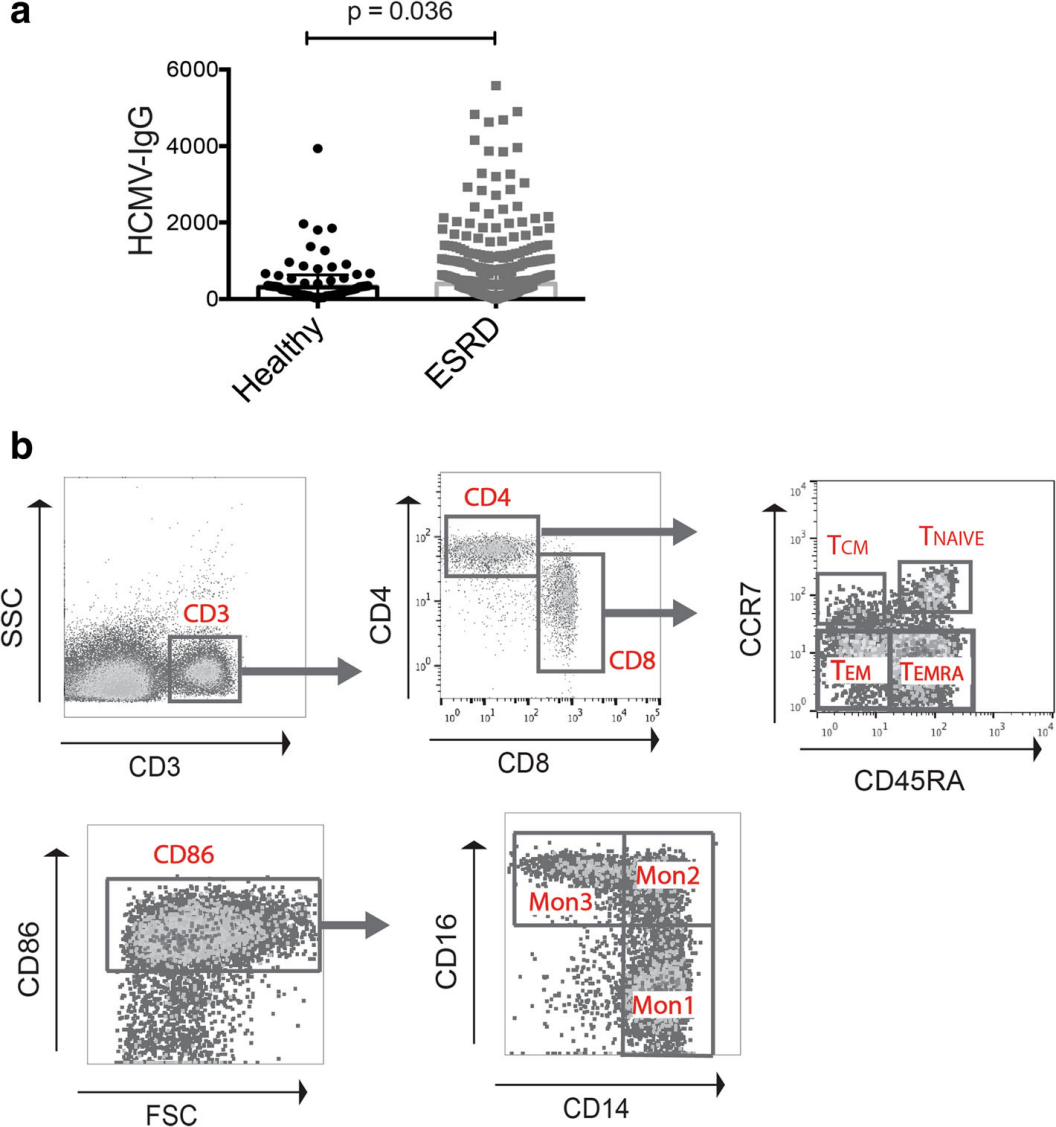
**a** Levels of HCMV-specific IgG (U/mL) were compared between healthy individuals and ESRD patients. The lines indicate medium and interquartile range. Statistical calculation of p value was performed using Mann-Whitney *U* non-parametric test. **b** Representative multicolor flow cytometry staining of T cell subsets (above) and monocyte subsets (below). T_NAIVE_, naïve T cells; T_CM_, central memory T cells; T_EM_, effector memory T cells; T_EMRA_, effector memory T cells with RA expression. Mon1, classical monocytes; Mon2, intermediate monocytes; Mon3, non-classical monocytes

First, we compared HCMV IgG levels between 408 CMV seropositive ESRD patients from the iESRD cohort and 57 CMV seropositive healthy individuals. The age of ESRD patients and healthy individuals were not statistically different (mean ± SD, ESRD: 62.0 ± 11.9; Healthy: 58.9 ± 6.8). Despite general belief of a more immunosuppressed state, ESRD patients exhibit significantly higher levels of HCMV IgG compared to healthy individuals (Fig. 1a, medium = 391.9 U/mL, IQR 181.5 ~ 818.5 versus medium = 305.8 U/mL, IQR 132.1 ~ 624.1). We further stratified HCMV IgG levels into quintiles and investigated the differences in demographic, clinical and laboratory data among groups. As Table 1 shows, patients in the highest HCMV IgG quintile tend to be older, have lower levels of hemoglobin, creatinine, phosphate and normalized protein catabolic rate, although most of these differences were not statistically significant.

**Table 1:** Baseline demographic, clinical and laboratory measurements stratified by HCMV-specific IgG titer

	1 st Quintile (*n* = 73)	2nd Quintile (*n* = 75)	3rd Quintile (*n* = 80)	4th Quintile (*n* = 87)	5th Quintile (*n* = 93)	*p* value
HCMV-IgG (U/ml)	76.9(35.4)	214.1(50.9)	367.6(48.5)	618.4(129)	1813(1079)	< 0.001*
Age (yr)	59.3(11.4)	62.9(13.4)	61.2(11.5)	62.4(12.0)	63.8(11.0)	0.16
Male (%)	56	49	51	52	46	0.79
Diabetes (%)	55	31	30	46	41	0.07
Dialysis vintage (yr)	4.7(3.5)	6.6(4.9)	6.1(4.7)	6.4(4.8)	6.7(6.2)	0.11
Albumin (g/dl)	4.1(0.33)	4.0(0.40)	4.1(0.30)	4.0(0.48)	4.0(0.42)	0.09
WBC (K/ul)	6.4(2.0)	6.2(2.0)	6.3(1.7)	6.6(2.2)	6.5(2.2)	0.75
Hemoglobin (g/dl)	11.1(1.3)	10.7(1.3)	11.3(1.1)	10.8(1.6)	10.6(1.4)	0.01*
BUN (mg/dl)	81.1(20.3)	79.5(20.3)	78.9(20.8)	79.5(20.6)	76.8(18.1)	0.72
Creatinine (mg/dl)	11.7(2.3)	11.1(2.7)	11.9(2.4)	10.7(2.4)	10.6(2.3)	0.001*
T-Cholesterol (mg/dl)	145(38.1)	151(34.0)	159(36.1)	154(42.6)	148(33.7)	0.15
Triglyceride (mg/dl)	148(93.9)	139(92.1)	146(100.3)	160(94.0)	143(92.1)	0.71
intact-PTH (pg/ml)	344(37.1)	405(46.9)	307(38.3)	308(37.8)	443(58.1)	0.12
Calcium (mg/dl)	9.2(0.7)	9.4(0.9)	9.4(0.7)	9.3(0.8)	9.4(0.8)	0.87
Phosphate (mg/dl)	5.2(1.5)	5.2(1.5)	5.1(1.2)	4.7(1.4)	4.6(1.3)	0.005*
Kt/V (Gotch)	1.35(0.2)	1.41(0.2)	1.40(0.2)	1.38(0.2)	1.39(0.2)	0.48
nPCR (g/Kg)	1.15(0.3)	1.23(0.4)	1.23(0.3)	1.21(0.3)	1.11(0.7)	0.40

Demographic and clinic data were compared between groups of CMV-IgG quintiles in 408 ESRD patients. Quintile cut-offs were derived from HCMV-IgG levels of healthy controls. Values were expressed as mean (SD)

*nPCR* normalized protein catabolic rate

*p value < 0.05

### Elevated HCMV IgG levels is not associated with systemic inflammation in ESRD

Since premature aging and systemic inflammation are important features of ESRD patients [31], we tested the associations between HCMV-specific IgG level with chronological age and circulatory inflammatory markers. We found a significant association between age and log-transformed HCMV IgG level (R = 0.15, *p* value = 0.003). Nevertheless, there was no relationship between HCMV IgG level and systemic inflammation, as measured by high-sensitivity C-reactive protein levels, TNFα levels and IL-6 (by Pearson correlation, all *p* value > 0.05). It has been suggested that elevated HCMV IgG level in asymptomatic individuals reflects virus reactivation and shedding [17]. As a result, augmented humoral response toward CMV in ESRD patients may be a unique immunological phenomenon that reflects aging and subclinical viral reactivation but not simply a status of non-specific systemic inflammation.

### Higher CMV-IgG level is associated with advanced T cell differentiation but not monocyte subset distribution in ESRD

Previous studies indicated that certain T cell and monocyte subsets [32–34] are associated with cardiovascular disease and/or atherosclerosis. Although it is known that CMV infection profoundly affects the adaptive human immune system [35, 36], much less is known about the effect of CMV infection on monocytes. We performed peripheral blood T cell and monocyte immunophenotyping in all the 408 CMV seropositive iESRD participants and tested the relationship between log-transformed HCMV-specific IgG level with distinct immune subsets, in either relative (percentage of mother population) or absolute (absolute cell number per μl of blood) terms. The representative multicolor flow cytometry staining is shown in Fig. 1b. Human T cells were separated into the CCR7+ CD45RA+ T_NAIVE_ subset, the CCR7+ CD45RA-T_CM_ subset, the CCR7-CD45RA-T_EM_ subset and the CCR7-CD45RAT_EMRA_ subset and the T_EM_ and T_EMRA_ subsets are known to increase in HCMV-infected individuals. As shown in Table 2, stronger humoral response against HCMV is associated with significant disturbance in the adaptive T cell homeostasis in ESRD. Patients with higher titer of HCMV-specific IgG exhibit lower percentages of CD4+ and CD8+ T cells but higher percentages of CD4 + CD28null and CD8+ terminally differentiated T_EMRA_ cells. Similar trends were observed when absolute cell number of each T cell subsets was analyzed and were also present in healthy controls (Additional file 1: Table S1). Higher titer of HCMV-specific IgG was associated with increased numbers of advanced differentiated CD4+ T cells. In contrast, HCMV-specific IgG level did not correlate with any specific monocyte subset changes. Previous studies in the literature only have compared T cell differentiation between HCMV seropositive and seronegative individuals. To our knowledge, this is the first description that the magnitude of humoral response against HCMV positively correlates with level of advanced terminal differentiation of both CD4+ and CD8+ T cells in ESRD patients. Overall, these results support the hypothesis that less-well controlled HCMV infection in ESRD patients drives the host adaptive immune system toward advanced terminal differentiation.

**Table 2:** Correlations between HCMV-specific IgG titer with levels of immune cells among ESRD patients

	Cell frequency		Absolute cell number	
	R	*p* value	R	*p* value
CD4+ T cells				
Naïve T cells	−0.33	NS	−0.05	NS
Stem Memory T cells	−0.03	NS	−0.12	NS
Central Memory T cells	−0.12	0.013*	−0.13	0.007*
Effector Memory T cells	0.14	0.006*	0.06	NS
Terminally Differentiated T cells	0.11	0.035*	0.010	0.003*
CD28 null cells	0.15	0.002*	0.12	0.02*
CD8+ T cells				
Naïve T cells	−0.15	0.003*	−0.18	< 0.001*
Stem Memory T cells	0.006	NS	−0.16	0.002*
Central Memory T cells	−0.10	0.039*	−0.15	0.003*
Effector Memory T cells	−0.03	NS	−0.03	NS
Terminally Differentiated T cells	0.20	< 0.001*	0.09	0.07
Monocytes				
Classical Monocytes	0.02	NS	0.04	NS
Intermediate Monocytes	0.01	NS	0.04	NS
Non-Classical Monocytes	−0.03	NS	−0.006	NS

Pearson correlation was applied to investigate the relationship between log transformed HCMV-specific IgG titer and immune cell levels, including percentages as well as absolute cell counts of naïve (T_NAIVE_), stem cell memory (T_SCM_), central memory (T_CM_), effector memory (T_EM_), terminally differentiated (T_EMRA_) subsets and three monocyte subsets (classical monocytes, intermediate monocytes, non-classical monocytes)

*NS* non-significant, *P* value > 0.1

*p value < 0.05

### Cardiovascular co-morbidities stratified by HCMV IgG levels

**Fig. 2:**
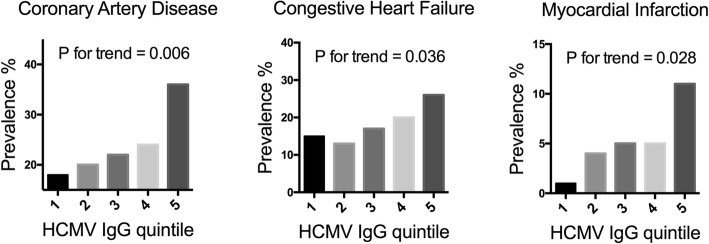
Percentage of patients with each specified co-morbidity among each HCMV-IgG quintile group is shown. Univariate regression analysis was performed to investigate the associations between IgG quintile and prevalence of individual co-morbidity

As reviewed in the introduction, previous studies performed on non-renal failure individuals have implicated HCMV infection in atherosclerotic vascular disease [37, 38]. As shown above, HCMV IgG level positively associates with the accumulation of terminally differentiated immunosenescent T cells. These cells have high atherogenic potentials, characterized by high CX3CR1 expression (thus allowing binding to injured endothelium) and capability of inducing endothelium damage [23, 24], eventually leading to atherosclerotic vascular diseases. These effector cells are likely to be HCMV-specific, and we previously have shown that HCMV-specific cells are of high degree of cytokine production as well as cytotoxic functions [39]. We thus compared the percentage of patients with various cardiovascular complications among patients belonging to different HCMV IgG quintiles. As shown in Fig. 2, patients within the highest IgG quintile had the highest prevalence of congestive heart failure (CHF), coronary artery disease (CAD), and history of old myocardial infarction. However, the prevalence of stroke (including both ischemic and hemorrhagic stroke) was not associated with higher of HCMV IgG levels (data not shown). When the relationship between HCMV IgG quintile and individual comorbidity were analyzed in regression models, higher IgG quintile was significantly associated with CAD (odds ratio = 1.25, *p* for trend = 0.006), CHF (odds ratio = 1.22, *p* for trend = 0.036), and history of MI (odds ratio = 1.48, *p* for trend = 0.014), but not with stroke (odds ratio = 0.84, *p* for trend = 0.19) and CVD (odds ratio = 1.13, p for trend = 0.32).

### HCMV IgG level independently associates with prevalent CAD

We next tested the independent association of HCMV-specific IgG level with CAD and CVD. Besides CAD, other individual co-morbidities were not tested because the percentages of patient with those co-morbidities were much lower. As shown in Table 3, higher HCMV-specific IgG quintile independently associated with prevalent CAD in both regression models, after adjusting for age, gender and other traditional and non-traditional cardiovascular risk factors. Nevertheless, the independent association between CVD and CMV IgG was not statistically significant. Similarly results were found when log-transformed IgG level was used in the regression model instead of HCMV IgG quintile (Additional file 1: Table S2).

**Table 3:** Association between HCMV-specific IgG quintile and coronary artery disease or cardiovascular disease

Variables in model (independent variable: CAD)	OR (95% CI)	*P* value
Model 1		
Age	1.03(1.01–1.05)	0.01*
Gender (Male)	1.43(0.90–2.29)	0.14
Diabetes	2.90(1.82–4.64)	< 0.001*
HCMV-specific IgG quintile	1.25(1.06–1.48)	0.007*
Model 2		
Age	1.04(1.01–1.06)	0.003*
Gender (Male)	1.39(0.86–2.25)	0.18
Diabetes	2.87(1.77–4.64)	< 0.001*
Albumin (g/dL)	1.44(0.67–3.12)	0.35
Hemoglobin (g/dL)	1.17(0.97–1.43)	0.10
Ca × P product (mg^2^/dL^2^)	1.01 (1.0–1.03)	0.27
hs-CRP (mg/dL)	1.37(1.12–1.67)	0.002*
HCMV-specific IgG quintile	1.27(1.07–1.51)	0.007*
Variables in model (independent variable: CVD)	OR (95% CI)	*P* value
Model 1		
Age	1.04(1.02–1.06)	< 0.001*
Gender (Male)	1.36(0.88–2.11)	0.17
Diabetes	2.88(1.86–4.48)	< 0.001*
HCMV-specific IgG quintile	1.13(0.97–1.32)	0.13
Model 2		
Age	1.03(1.02–1.06)	< 0.001*
Gender (Male)	1.32(0.84–2.08)	0.22
Diabetes	2.84(1.80–4.44)	< 0.001*
Albumin (g/dL)	1.07(0.52–2.16)	0.87
Hemoglobin (g/dL)	1.20(1.00–1.44)	0.048*
Ca × P product (mg^2^/dL^2^)	1.01 (0.99–1.03)	0.32
hs-CRP (mg/dL)	1.32(1.08–1.60)	0.005*
HCMV-specific IgG quintile	1.13(0.96–1.33)	0.13

Multivariable-adjusted logistic regression models, including age, gender, diabetes mellitus, albumin, hemoglobin, calcium phosphate product and high sensitivity-CRP were used to investigate the independent association between HCMV IgG quintile and co-morbidities. *: *p* value < 0.05

## Discussion

In the general population, HCMV infection is related to many adverse clinical conditions; atherosclerotic vascular complications are by far one of the best studied [37, 40]. In this study, we successfully demonstrated the association between higher anti-HCMV IgG titer in ESRD patients and higher risk for CAD, and suggested a potential mechanistic link between subclinical HCMV reactivation, aggravated T cell effector differentiation and coronary artery disease in this patient population. Our results indicate that it is necessary to continue investigating the long-term impact of immune response against HCMV in ESRD in longitudinal studies.

ESRD patients are characterized by a marked status of chronic systemic inflammation [41]. Interestingly, although HCMV IgG titer does not correlate with systemic inflammation in our study, previous studies indicate persistent HCMV infection has stronger impact in individuals with chronic inflammation. In a study involving 989 non-renal patients with CAD [42], HCMV seropositivity was independently associated with a 3.2-fold increase in risk of future cardiac death only in patients with high IL-6 levels, whereas in individuals without IL-6 elevation, HCMV had no effect on cardiac mortality. Another study showed that HCMV seropositivity in combination with elevated hsCRP is a strong, independent predictor of future cardiac death [38]. Because chronic inflammation is a pertinent feature of renal failure patients, ESRD patients might be prone to suffer from HCMV-associated adverse effects on cardiovascular complications.

Our study also found that HCMV-specific IgG level positively correlated with advanced T cell differentiation. Cantisan et al. had found that the loss of CD27 and CD28 on HCMV-specific T cells post solid organ transplantation correlated with HCMV replication and this process was age-dependent [43]. Advanced T cell differentiation has been found in ESRD seropositive for HCMV [44], and ESRD patients also demonstrate a shift in their T cell receptor Vβ chain repertoire [45], which is correlated with age. It remains unknown if level of HCMV-specific IgG correlates with Vβ diversity in ESRD patients.

Only few cohort studies have investigated the role of HCMV-specific IgG in cardiovascular mortality in the general population. In the population-based Atherosclerosis Risk in Community (ARIC) study, people with the highest level of anti-HCMV IgG exhibited a 1.76-fold higher risk for coronary artery disease during a five-year follow-up when compared to the lowest level group [16]. People with diabetes are also affected by HCMV infection, with a relative risk of 9.2. In another population-based study (EPIC-Norfolk), individuals with the highest level of anti-HCMV IgG also had a 1.22-fold higher risk for ischemic heart disease when compared to seronegative individuals [15]. Our current study in ESRD patients indicates elevated HCMV IgG titer is related to coronary artery disease and history of myocardial infarction, but we did not find significant association between IgG titer and stroke. Interestingly, while CMV viral DNA is frequently found in coronary artery atherosclerotic plaques, some studies had reported the lack of CMV DNA in carotid artery plaques [46].

The exact mechanisms by which persistent HCMV infection results in higher cardiovascular mortality remain elusive [47], but the accumulation of terminally differentiated T cells could be a plausible mechanism. Our recent studies showed anti-HCMV IgG level positively correlates with the total size of virus-specific T cell pool [39] and inversely correlated with T cell receptor diversity [48] in individuals without renal disease. A recent report further showed that HCMV-specific senescent T cells are associated with arterial stiffness [49]. After adherence to activated vascular endothelium via CXCR3, these cytotoxic T cells further participate in atherogenesis by directly causing endothelial damage [24] and they also secrete high level of TNFα [39], the critical cytokine to activate macrophages. Intermediate monocytes (CD14++CD16+) are known to participate in atherosclerosis [50] but we discovered that HCMV-specific IgG did not affect the level of intermediate monocyte, indicating a different mechanism for monocyte activation in ESRD patients. Since our study did not measure the extent of atherosclerosis or arteriosclerosis, we could not analyze the impact of immune response on these pathological parameters separately. Animal studies of MCMV infection in renal failure mice will help to provide more detailed information.

There are additional limitations with this study. First, because the study is built on cross-sectional data only, we could not yet establish the causal relationship between levels of anti-HCMV IgG and cardiovascular complications. In addition, it remains technically difficult to detect subclinical HCMV reactivation, as attempts to analyze serum viral load turned out negative for all of our patients (data not shown). As a result, the mechanism for variable HCMV-specific IgG titers in ESRD patients is still unknown. Finally, it remains unknown if HCMV-specific IgG level is dynamic or stable in ESRD patients and follow-up data from our ongoing iESRD cohort will provide insight into this question.

In conclusion, our current study is the first to demonstrate that anti-HCMV IgG titer is elevated in ESRD patients with persistent HCMV infection and associates with prevalent coronary artery disease. The effect is significant and independent of traditional or non-traditional cardiovascular risk factors, including inflammation. Level of HCMV IgG also positively correlates with T cell terminal differentiation, which could serve as the mediator for this association. This implies a pathogenic role of HCMV reactivation in ESRD patients, and further studies are warranted to continue investigating the long-term effects of persistent HCMV infection in this susceptible population.

## Conclusions

Our study indicates that anti-HCMV specific IgG is elevated in ESRD patients, who are at high risk of CAD. The elevation of HCMV-specific IgG positively correlates with advanced T cell differentiation but does not with monocyte subset homeostasis. In ESRD patients, HCMV-specific IgG level is independently associated with prevalent of CAD. The impact of persistent HCMV infection on CAD should be further investigated in this patient population.

## Additional file

Additional file 1: **Table S1.** Correlations between HCMV-specific IgG titer with levels of immune cells among healthy individuals. **Table S2.** Associations between log-transformed HCMV-specific IgG level with coronary artery disease and cardiovascular disease. (DOCX 26 kb)

## Abbreviations

CAD: Coronary artery disease
CHF: Congestive heart failure
CVD: Cardiovascular disease
ESRD: End-stage renal disease
HCMV: Human cytomegalovirus
