# Supplementary material for: Anti-cytomegalovirus IgG antibody titer is positively associated with advanced T cell differentiation and coronary artery disease in end-stage renal disease
Source: Immun Ageing. 2018 Jul 2;15:15. doi: 10.1186/s12979-018-0120-0 (PMC6029034; doi:10.1186/s12979-018-0120-0)
Supplement: Supplementary file 1 — Table S1. Correlations between HCMV-specific IgG titer with levels of immune cells among healthy individuals. Table S2. Associations between log-transformed HCMV-specific IgG level with coronary artery disease and cardiovascular disease. (DOCX 26 kb) [file 12979_2018_120_MOESM1_ESM.docx]

**Table S1**

**Correlations between HCMV-specific IgG titer with levels of immune cells among healthy individuals**

|  | - Cell frequency | | - Absolute cell number | |
| --- | --- | --- | --- | --- |
|  | - R | - P value | - R | - P value |
| - CD4+ T cells |  |  |  |  |
| - Naïve T cells | - -0.17 | - NS | - -0.05 | - NS |
| - Stem Memory T cells | - 0.10 | - NS | - 0.08 | NS |
| - Central Memory T cells | - -0.02 | NS | - -0.12 | - NS |
| - Effector Memory T cells | - 0.19 | NS | - 0.16 | NS |
| - Terminally Differentiated T cells | - 0.24 | - 0.07 | - 0.28 | - 0.03* |
| - CD28 null cells | - 0.30 | - 0.02* | - 0.39 | 0.003* |
|  |  |  |  |  |
| - CD8+ T cells |  |  |  |  |
| - Naïve T cells | - -0.21 | - NS | - -0.16 | NS |
| - Stem Memory T cells | - 0.11 | NS | - 0.13 | - NS |
| - Central Memory T cells | - 0.03 | - NS | - 0.14 | - NS |
| - Effector Memory T cells | - 0.08 | - NS | - 0.22 | - NS |
| - Terminally Differentiated T cells | - 0.21 | - NS | - 0.27 | - 0.047* |
|  |  |  |  |  |
| - Monocytes |  |  |  |  |
| - Classical Monocytes | - -0.24 | - 0.07 | - 0.24 | - 0.07 |
| - Intermediate Monocytes | - -0.03 | - NS | - 0.04 | - NS |
| - Non-Classical Monocytes | - 0.13 | - NS | - 0.25 | - 0.06 |

Pearson correlation was applied to investigate the relationship between log transformed HCMV-specific IgG titer and immune cell levels, including percentages as well as absolute cell counts of naïve (T_NAIVE_), stem cell memory (T_SCM_), central memory (T_CM_), effector memory (T_EM_), terminally differentiated (T_EMRA_) subsets and three monocyte subsets (classical monocytes, intermediate monocytes, non-classical monocytes). NS: non-significant with P value > 0.1. *: P value < 0.05.

**Table S2**

**Associations between log-transformed HCMV-specific IgG level with coronary artery disease and cardiovascular disease**

| - Variables in model - (independent variable: CAD) | OR (95% CI) | - P value |
| --- | --- | --- |
| - Model 1 |  |  |
| - Age | - 1.03(1.01-1.05) | - 0.016* |
| - Gender (Male) | - 1.45(0.91-2.32) | - 0.12 |
| - Diabetes | - 2.97(1.86-4.74) | - <0.001* |
| - log HCMV-specific IgG | - 1.95(1.20-3.16) | - 0.007* |
| - Model 2 |  |  |
| - Age | - 1.03(1.01-1.05) | - 0.004* |
| - Gender (Male) | - 1.41(0.88-2.28) | - 0.16 |
| - Diabetes | - 2.84(1.76-4.59) | - <0.001* |
| - Albumin (g/dL) | - 1.46(0.68-3.14) | - 0.36 |
| - Hemoglobin (g/dL) | - 1.18(0.98-1.44) | - 0.10 |
| - Ca×P product (mg^2^/dL^2^) | - 1.01 (1.0-1.03) | - 0.27 |
| - hs-CRP (mg/dL) | - 1.36(1.11-1.65) | - 0.002* |
| - log HCMV-specific IgG | - 2.01(1.21-3.33) | - 0.007* |
| - *Variables in model* - *(independent variable: CVD)* | - *OR (95% CI)* | - *P value* |
| - Model 1 |  |  |
| - Age | - 1.03(1.01-1.06) | - 0.001* |
| - Gender (Male) | - 1.04(0.88-2.13) | - 0.16 |
| - Diabetes | - 2.93(1.88-4.55) | - <0.001* |
| - log HCMV-specific IgG | - 1.45(0.93-2.26) | - 0.11 |
| - Model 2 |  |  |
| - Age | - 1.04(1.02-1.06) | - <0.001* |
| - Gender (Male) | - 1.34(0.85-2.10) | - 0.21 |
| - Diabetes | - 2.81(1.79-4.40) | - <0.001 |
| - Albumin (g/dL) | - 1.08(0.53-2.18) | - 0.83 |
| - Hemoglobin (g/dL) | - 1.20(1.01-1.43) | - 0.048* |
| - Ca×P product (mg^2^/dL^2^) | - 1.30 (1.09-1.53) | - 0.32 |
| - hs-CRP (mg/dL) | - 1.31(1.08-1.59) | - 0.006* |
| - log HCMV-specific IgG | - 1.46(0.82-2.32) | - 0.14 |

Multivariable-adjusted logistic regression models, including age, gender, diabetes mellitus, albumin, hemoglobin, calcium phosphate product and high sensitivity-CRP were used to investigate the independent association between log-transformed HCMV IgG level and co-morbidities.
